# Quantitative Model of Multiple Crystal Growth Rate Minima in Polymers with Regularly Spaced Substituent Groups

**DOI:** 10.1021/acs.macromol.3c02432

**Published:** 2024-02-12

**Authors:** Kutlwano Gabana, Gillian A. Gehring, Xiangbing Zeng, Goran Ungar

**Affiliations:** † Department of Physics and Astronomy, University of Sheffield, Sheffield S3 7RH, U.K.; ‡ Department of Materials Science and Engineering, University of Sheffield, Sheffield S1 3JD, U.K.; § Shaanxi International Research Center for Soft Materials, School of Material Science and Engineering, Xi’an Jiaotong University, Xi’an 710049, China

## Abstract

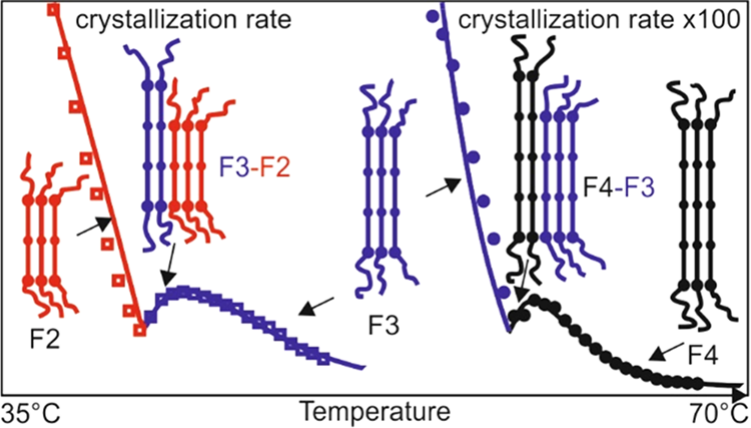

A simple theory has been developed to explain quantitatively the multiple crystal growth rate minima observed experimentally in polyethylene brassylates (PEBs), polymers with regularly spaced “chemical defects”, in this case, diester groups separated by 11 methylenes. The minima occur at the transitions where the fold length drops from 4 to 3 repeat units and from 3 to 2 units. An analytical rate-equation model was developed with elementary attachment and detachment steps of individual monomer repeat units, also including postattachment stem lengthening (stem conversion). The model produced a good fit to experimental crystallization rate curves for PEBs of three different molecular weights. The fits confirm in a quantitative way that the anomalies are caused by the self-poisoning effect, as proposed in the original experimental report on PEBs, based on the ideas developed in previous studies on long-chain *n*-alkanes. It is concluded that the rate minima in PEBs are the result of temporary attachment to the growth surface of stems that are too short to be stable yet long enough and close to stability to obstruct productive growth by stems of sufficient length. The results confirm the ubiquitous presence of self-poisoning at the growth front of polymer crystals in general and will help to achieve a better understanding of the complex process of crystallization of polymers. It will also allow the determination of more realistic parameters controlling their lamellar growth kinetics.

## I Introduction

Recently, crystal growth rate minima have been reported as a function of crystallization temperature by Alamo and co-workers in several polydisperse polyethylenes in which replacement atoms or groups had been placed at equal distances along the chain. Where the substituents were bromine atoms, a growth rate minimum was found at the transition between two different crystal polymorphs of the polymer.^1^ Similar behavior has also been reported in precision polyacetals.^2^ In polyethylene brassylate (PEB), 
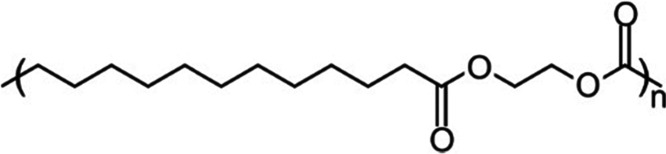
, with a double ester group spaced by 11 CH_2_ units, two growth rate minima were observed in three polymers of different molar mass. These occur at transitions between different preferred lamellar thicknesses within the same crystal form. Lamellar thickness is quantized since the diester group is a preferred fold site. Therefore, a polymer stem (a segment between two folds in the crystalline layer) always consists of an integer number of repeating units.^3^

In PEB, the observed growth rate minima bear many similarities with those in monodisperse long-chain alkanes with 100–400 carbons, whose melt-crystallization rate was found to pass through a minimum with increasing supercooling.^4,5^ An even more pronounced effect was found in their crystal growth from solution.^6^ The minimum was accompanied by drastic changes in crystal shape.^7^ From the analysis of curvature of lateral crystal faces, it was established that at the minimum, the rate of initiation of new layers of stems, *i*, was suppressed more than the rate of the layer propagation, *v*.^8^ A clear, though less dramatic rate minimum was also observed in melt-crystallization of narrow low-molecular-weight fractions of poly(ethylene oxide) (PEO).^9^ Contrary to alkanes, curvature analysis in PEO showed the minimum in *v* to be deeper than the minimum in *i*.^10^

It has also been observed in long alkanes that growth rate at constant temperature from solution passes through a very deep minimum, of zero growth, with increasing solution concentration.^11^ These anomalies caused other unusual effects such as the “dilution wave” spreading through a metastable supersaturated solution triggering an avalanche-like wave of precipitation.^12^

**Figure 1:**
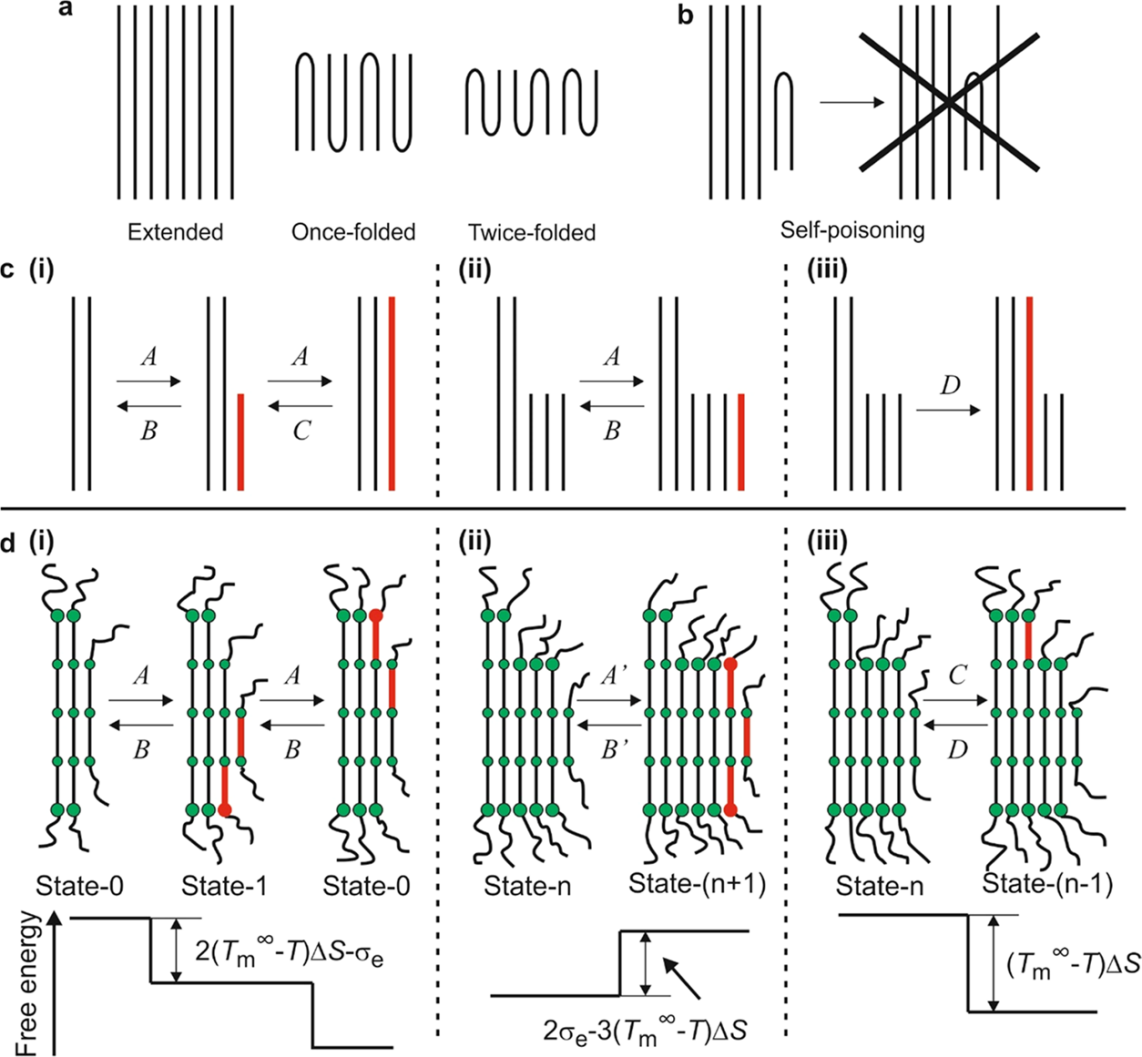
Self-poisoning in long-chain *n*-alkanes and in PEB. (a) Integer folded forms of long-chain *n*-alkanes, where molecules are folded exactly in two or three with the tight chain folds and chain ends always at the surfaces of the crystalline layers. (b) The growth of the extended form can be “poisoned” by the frequent attachment of the unstable once-folded chains at the growth front, preventing further attachment and the growth of the extended form.^15^ (c) Previous growth model of the extended-chain form with self-poisoning effect accounted. (d) Schematic depiction of the growth steps in PEB. The circles represent the double ester units. The free energy difference between different states is shown in their schematics. Note that for simplicity, the amorphous lamellar surface is shown as containing only chain ends, ignoring the chain folds. (i) Growth of F4 via intermediate formation of an F3 stem at the growth front. Note the last F4 stem in State-0 is automatically covered by an F2 stem, as such stems do not require the formation of new overcrowded end surface. The rate-limiting steps in the growth of an F4 stem are crystallization of the two end segments with the forward rate *A* and the backward rate *B*. The first step leads to the formation of an intermediate F3 stem at the growth front (State-1); with crystallization of the other end segment, the system again returns to State-0. (ii) Formation of a sequence of F3 stems at the growth front, with the deposition rate of a further F3 stem as *A′* (State-*n* to State-(*n* + 1), *n* > 1), and the reverse detachment rate *B*′ (State-(*n* + 1) to State-*n*, *n* > 1). (ii) A covered F3 stem at the growth font of F4 can convert to an F4 stem with conversion rate *C*, significantly lower than *A*, and with a still lower back-conversion rate *D*.

The long-chain *n*-alkanes that show such anomalous crystallization form lamellar crystals with thickness/fold length an integer fraction of the contour length of the molecule. In such crystals, the molecules are either extended or folded exactly in two, three, four ··· with all chain folds and ends at the surface of the lamellae (Figure 1a).^13^ Growth and nucleation rate minima were found at transitions between different integer folded forms, up to three-times folded in *n*-C_390_H_782_,^14^ and the concept of “self-poisoning” was first introduced in 1987.^4^ It is believed that just above the growth rate minimum, there are frequent attachments to the growth front of “nearly stable” integer folded chains. For example, a once-folded stem can attach to the surface of a growing extended-chain crystal (Figure 1b), and when the temperature is still above the melting point of the folded chain form, the folded chain attachments will not be able to grow.^15^ However, further growth of the extended-chain form can proceed only when the folded chain attachment has been removed from the growth surface. This is similar to the “poisoning” effect in crystal growth, where the growth of a crystal is hampered by the presence of impurities on the growth front. However, in self-poisoning, the “poison” is not an impurity but a host molecule in a wrong conformation.

Reproducing the growth rate minima is a good test of the validity of different polymer crystallization theories. Thus, while classic coarse-grain Lauritzen–Hoffman (LH) theory could not reproduce them without some strained assumptions,^16^ with fine-grain models such as Sadler’s roughness-pinning theory, the minima appear naturally, at least qualitatively.^17^ Similarly, Monte Carlo simulation based on segmented polymer chains was also able to show the minima, even though less pronounced than in real alkane systems.^18^ More recently, based on mean-field theory and computer simulation, it was argued that self-poisoning is ubiquitous, as long as a molecule can bind in two (or more) energetically nonequivalent ways to a crystal, and their binding probability is sufficiently different.^19^ Recently, the self-poisoning effect has also been reported in polyglutamine, a polypeptide sequence that causes Huntington’s and other amyloid-associated neurodegenerative diseases, where amyloid formation does not monotonically increase with increasing concentration of the polypeptide.^20^

It is therefore important to emphasize that self-poisoning is not just a freak anomaly of monodisperse model compounds or polymers with regularly spaced “defects” but that it is an intrinsic feature of crystallization of flexible polydisperse polymers. At a crystallization temperature, there is a minimum stem length *l*_min_ below which crystal growth cannot proceed. However, attached stems that are slightly shorter are expected to have a sufficiently long lifetime to obstruct growth of lamellae with *l* > *l*_min_. This has been clearly shown in Monte Carlo^21^ and molecular dynamics simulation of lamellar growth in polyethylene.^22^ In polydisperse polymers, the growth or nucleation rates normally increase with increasing supercooling without any minima, as the lamellar thickness changes continuously with supercooling. Were it not for self-poisoning, polymer crystallization would have proceeded much faster than experimentally observed. In classical theories, the retardation due to self-poisoning is subsumed in the pre-exponential viscosity term.

The crystal growth rate minima in PEB have been qualitatively attributed to self-poisoning, implying that the growth of a stable lamellar structure with longer fold length was poisoned by unstable but frequent attachment of polymer chains of shorter fold length at the growth front.^3^ In the current work, we aim to develop a quantitative model to explain the growth rate minima in PEB. Such a model will also help us to understand better the complex polymer crystallization process and refine the main parameters controlling their growth kinetics.

## II Model

Higgs and Ungar^11,15^ developed a model that was able to semiquantitatively explain the growth rate minimum at the transition between extended and once-folded forms of long-chain *n*-alkanes. It was assumed that the growth of the extended form at the growth front is by two consecutive steps of attachment of half of a stem, each with the same attachment rate, *A*. Above the melting point of the once-folded form, a half-crystallized stem at the growth front is unstable so its detachment rate, *B*, is larger than *A*, but the extended stem at the growth front is stable and the rate of it reverting back to a half-crystallized stem, *C*, is smaller than *A* (Figure 1c,i). The once-folded chains at the growth front, even though unstable, can further grow in numbers by attachment of more half-chains, but an extended stem can grow only when such additional half-chains are removed (Figure 1c,ii). Closer to the melting point of the once-folded form, the length of the half-chains attaching to the growth front increases. Consequently the growth rate of the extended form is poisoned, and in fact, according to the model, the growth rate of the extended form would drop to zero at the melting point of the once-folded form. A nonzero growth rate minimum can be generated when a covered half-crystallized molecule is allowed to convert to the extended form (conversion rate *D*) at the extended stem growth front (Figure 1c,iii).

In this paper, we have followed the same concept and modified the Higgs–Ungar (H–U) model to explain the growth rate minima observed in PEBs. In the H–U model, just breaking the stem into two half-stems allowed it to, semiquantitatively, reproduce the growth rate minimum at the transition from extended to once-folded chain growth, a task unachievable by the coarse-grain LH theory. However, in order to tackle the crystallization kinetics of segmented polymers like PEB, we had to go further toward a fine-grain theory and split the stem into individual monomer units. In fact, further “fine-graining” was shown to be necessary, not surprisingly as each monomer repeat still contained a number of flexible bonds. However, in this paper, we chose not to split the segments completely into individual methylene groups but instead used a shortcut of making the segment attachment rate temperature-dependent (see below). We then used the model to fit the published experimental growth rate curves, which allowed us to derive some parameters governing the actual crystallization of PEB.

Regularly patterned polyethylene brassylates will naturally crystallize with a crystalline layer thickness corresponding to *N* repeating units, where *N* is an integer. This is to allow for a smaller surface energy of the crystals where the ester groups can stay at the crystal–amorphous interface where the chain folds. For convenience, in this paper, we will refer to these as *N*-unit form, as the crystalline layer of each lamella consists of *N* sublayers, whose thickness is the length of a repeating unit. For simplicity, hereafter, we will use the abbreviation “F*N*” to stand for the *N*-unit form and for *N*-unit stems, the abbreviation F*N* stems. The melting temperature of such lamellae will depend on the crystalline layer thickness, as in other semicrystalline polymers, i.e., a higher value of *N* will lead to a higher melting temperature *T*_m,*N*_.

It should be noted here that for simplicity, like the H–U model, our model considers the crystal growth as a one-dimensional (1D) process; therefore, the effects of lateral surface energy and lateral growth are ignored.

The growth steps of our model are shown in Figure 1d, where the growth of F*N* is poisoned by the competing growth of F(*N* – 1). We consider the growth of the two forms in a temperature region, where F*N* grows at higher temperatures and F(*N –* 1) at lower temperatures. The melting temperatures of the two forms are *T*_m,*N*_ > *T*_m,*N*–1_, and the self-poisoning effect is expected to be most significant close to *T*_m,*N*–1_.

In Figure 1d, the diagrams are drawn for *N* = 4, and in the following, the model will be explained with *N* = 4 as an example. In the high-temperature range, the ways in which F4 can grow are schematically shown in Figure 1d,i, where the molecular segments (stems) in the crystalline layer are shown as straight lines. The double ester units on the crystalline stems are represented by circles.

We consider the rate-limiting step for the growth of a polymer stem to be the formation of the crystalline–amorphous layer interface, i.e., the end- or fold-surface of each deposited stem at the growth front. Overcrowding of the amorphous stem ends at the interface causes either a reduction in the entropy of the amorphous stems at the interface or an increase in energy by way of tight stem folding.^23−26^ We have made the ester units at the interfaces bigger than those inside the crystalline layer in Figure 1d, to show that each of them carries an extra surface energy σ_e_. A consequence of our assumption is that an F4 stem at the growth front is readily covered with an F2 stem because an F2 stem can avoid having either of its end groups in the overcrowded state and would therefore incur no additional end-surface free energy penalty (State-0 in Figure 1d,i). Here, the state of the growth front is recorded as State-*n*, with *n* being the number of F3 stems at the front.

Starting with State-0, the growth of F4 by one stem is realized in two steps. In the first step, a new end-surface site is created by the addition of an end unit to the F2 stem at the growth front, making it an F3 stem. At the same time, another F1 stem is readily deposited on the F3 stem (State-1, Figure 1d,i, with the newly grown units shown in red). Half of an F4 stem is therefore grown with two repeating units newly crystallized and one new stem-wide end-surface patch created. In the second step, the F3 stem grows into an F4 stem; thus, the growth front returns to state-0 after advancing by one stem width. Like the first step, half of an F4 stem is crystallized along with one end-surface patch. When the temperature is below the melting point of F4 (*T*_m,4_), both steps combined would reduce the system free energy by the same amount 2(*T*_m_^∞^ – *T*)Δ*S* – σ_e_, where *T*_m_^∞^ is the ultimate melting temperature of infinitely thick crystals and Δ*S* is the melting entropy per repeat unit. The kinetics of the two steps, State-0 → State-1 and State-1 → State-0, are assumed to be exactly the same, with the forward rate *A* and the backward rate *B* as shown in Figure 1.

As long as the crystallization temperature is well above the melting point of the three-unit form *T*_m,3_, no other growth steps are needed to describe the growth of F4. However, at temperatures closer to *T*_m,3_, a temporary buildup of F3 stems at the growth front must be considered as shown in Figure 1d,ii. Such F3 stems at the growth front, even though unstable above *T*_m,3_, will hamper or poison the growth of F4, as in state State-*n* where *n* > 1, no direct deposition of a new F4 stem at the growth front is possible. The transformation from State-*n* to State-(*n* + 1), by the addition of another F3 stem to the growth front, has rate *A*′, and the reverse process has rate *B*′. The associated free energy change is 3(*T*_m_^∞^*– T*)Δ*S* – 2σ_e_. Above *T*_m,3_, this free energy change is positive, F3 stems are unstable and *B*′ > *A*′.

Another growth step that should be considered is shown in Figure 1d,iii. A covered F3 stem in State-*n*, where *n* > 1, immediately in contact with F4 stems, can convert to an F4 stem and contribute to the crystal growth. In the process, one repeating unit is crystallized, and there is no creation of an additional overcrowded end-surface patch. The free energy is reduced by (*T*_m_^∞^ – *T*)Δ*S*. The conversion rate is *C*, and the reverse process has a rate *D*. As the barrier for the conversion of a covered 3-unit stem is expected to be high, *C* should be significantly smaller than *A*.

We define the probabilities of having a clean F*N* surface (State-0) as *P*_0_, and those of having surfaces with 1, 2, 3, ···, *n* F(*N* – 1) stems (State-1, 2, 3··· *n*) attached as *P*_1_, *P*_2_, *P*_3_··· *P*_*n*_. Like in previous studies, we employ a steady-state growth model that means *P*_*n*_ does not change with time so that we have the conditions
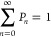
1and
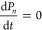
2The growth equations and the steady states solutions are given as follows:
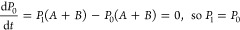
3a
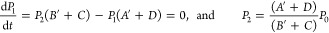
3b
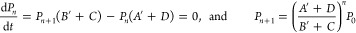
3cThe steady-state growth condition of *N*-unit form requires that
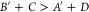
4Combining eqs 1 and 3a–c, we have
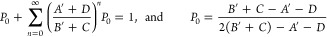
5F*N* grows because of the attachment *A* (between State-0 and State-1) and the conversion *C* (State-*n* to State-(*n* – 1), for *n* ≥ 2) overcoming the detachment rates *B* (between State-0 and State-1) and *D* (State-*n* to State-(*n* + 1), for *n* ≥ 1). Therefore, the growth rate of the F*N* form can be derived as
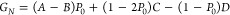
6At lower temperatures, the growth of F(*N* – 1) overtakes that of F*N*. Its growth rate is
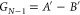
7The transition between F*N* and F(*N –* 1) growth occurs at the temperature (poisoning temperature) where *B*′ + *C* = *A*′ + *D* so *P*_*n+*1_ = *P*_*n*_. The experimentally observed growth rate will be given by the larger of *G*_*N*_ and *G*_*N*–1_.

Direct measurement of growth rates of spherulites of PEBs with different molar masses by optical microscopy was limited to the F4 form and only to a few degrees below the growth transition (rate minimum) to the F3 form. With further supercooling, the growth rate of F3 becomes too fast to be measured accurately by microscopy.^3^ Instead the crystallization rates across the F4, F3 and F2 temperature regions have been measured by calorimetry, specifically differential scanning calorimetry (DSC) in the F4–F3 range and flash scanning calorimetry (FSC) in the F3–F2 range. The inverse of the time for the polymer to release half of its crystallization enthalpy (1/*t*_0.5_) was used as the measure of the crystallization rate. Even though these are not direct measurements of the growth rate, we take the assumption that the crystallization rate measured is proportional to the growth rate as defined above and used the DSC/FSC data in the fittings detailed in the following, using eqs 1–7. This assumption is supported by the similarity in the shapes of the crystallization rate curves from DSC/FSC and the growth rate curves directly measured by microscopy for the same polymer.^3^

## III Estimation of the Parameters

The relationship between the three pairs of parameters, *A* and *B*, *A*′ and *B*′, and *C* and *D*, can be established based on the free energy difference between related states, which are shown in Figure 1d.

The free energy difference between states State-0 and State-1 comes from the free energy of crystallization of half of an *N*-stem, and the formation of a new end-surface patch with a free energy cost σ_e_. Assuming the ultimate melting temperature of the polymer (without chain folding) to be *T*_m_^∞^ and the entropy of melting for each repeating unit Δ*S*, the free energy of crystallization of each repeating unit at temperature *T* should be (*T*_m_^∞^ – *T*)Δ*S*. At each forward step in Figure 1d,i, the free energy is reduced by
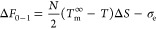

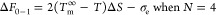
8Therefore, we have
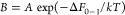
9The melting point of F*N* form (*T*_m,*N*_) can be derived under condition that *A* = *B*
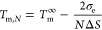
10The free energy of crystallization of an F(*N* – 1) stem in Figure 1d,ii is
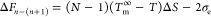

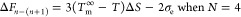
11and
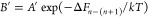
12The conversion of an F(*N* – 1) stem to an F*N* stem does not involve the formation of a new end surface, so
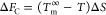
13and
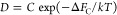
14The important fitting parameters *T*_m_^∞^, Δ*S*, and σ_e_ are linked to the melting points of F4, F3, and F2 by eq 10. Therefore, their chosen values must be consistent with experimental observations (a) that the highest temperature at which growth of F4 is observed is ∼69 °C (this should be close to *T*_m,4_) and (b) that the two growth rate minima (F4–F3 and F3–F2) are observed at 58–60 °C (should be slightly below *T*_m,3_) and 40–43 °C (slightly below *T*_m,2_), respectively.^3^ The ultimate melting points *T*_m_^∞^ of PEBs of different molar masses were adjusted to be as close as possible. At the same time, it was ensured that the resulting *T*_m,4_, *T*_m,3_, and *T*_m,2_ values were consistent with the experimental observations while providing the best fit to crystallization rate data. The best-fit ultimate melting temperatures of the polymers are the same (and so are *T*_m,4_, *T*_m,3_, and *T*_m,2_) for PEB90 and PEB188 (95.4 °C) and only slightly lower for PEB27 (95.0 °C) (Table 1). The slightly lower melting point of PEB27 may be linked to the increased melt entropy due to its higher proportion of chain ends.^27^

**Table 1:** Melting Enthalpy Δ*H* (Values Taken from Reference (3)) and Melting Entropy Δ*S* of One Repeating Unit, Surface Energy σ_e_, Best-Fit Melting Points of Polymers with Infinite Fold Length (*T*_m_^∞^) and F4 (*T*_m,4_), F3 (*T*_m,3_), and F2 Forms (*T*_m,2_) Used in the Fitting of Experimental Crystallization Data

	Δ*H* (J g^–1^)	Δ*S* (J K^–1^ mol^–1^)	σ_e_ (J mol^–1^)	*T*_m_^∞^ (°C)	*T*_m,4_ (°C)	*T*_m,3_ (°C)	*T*_m,2_ (°C)
PEB27	82.3	121	6230	95.0	68.0	59.0	41.0
PEB90	70.8	104	5600	95.4	68.9	60.0	42.0
PEB188	90.6	132	7150	95.4	68.9	60.0	42.0

The melting entropy Δ*S* of each repeating unit can be estimated from the measured mass melting enthalpy Δ*H* (Table 1, assuming a 0.50 crystallinity), using the equation
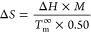
15Here, *M* is the molar mass of the repeat unit of the polymer, 270.36 g mol^–1^. The differences in the values of Δ*S* derived for PEB27, PEB90, and PEB188 can be attributed to the variation in the difference between their actual crystallinity and the assumed value of 0.50. Thus, PEB90 has the lowest experimentally measured crystallinity by wide-angle X-ray scattering (WAXS) (0.39–0.44 depending on crystallization temperature), while that for PEB188 is 0.50–0.59.^3^ An averaged  value of 119 J K^–1^ mol^–1^ and similarly an averaged end-surface energy  of 6327 J mol^–1^ are used in subsequent data fitting.

The entropy of melting of PEB can also be compared to those of poly(butylene succinate) (PBS) and polyethylene. The melting point of PBS (molar mass 172.18 g mol^–1^) is 114.1 °C, with heat of fusion 68.4 J g^–1^ and crystallinity of 0.60, resulting in an entropy of melting of 50.7 J K^–1^ mol^–1^ (of butylene succinate repeating unit).^28^ At the same time, polyethylene has an ultimate melting point of 419 K and heat of fusion of 4100 J mol^–1^, with entropy of melting of CH_2_ 9.79 J K^–1^ mol^–1^. As PEB has seven more CH_2_ groups compared to PBS, a simple extrapolation (50.7 + 9.79 × 7) would result in a melt entropy of 119.2 J K^–1^ mol^–1^, almost exactly the same as estimated above.

**Figure 2:**
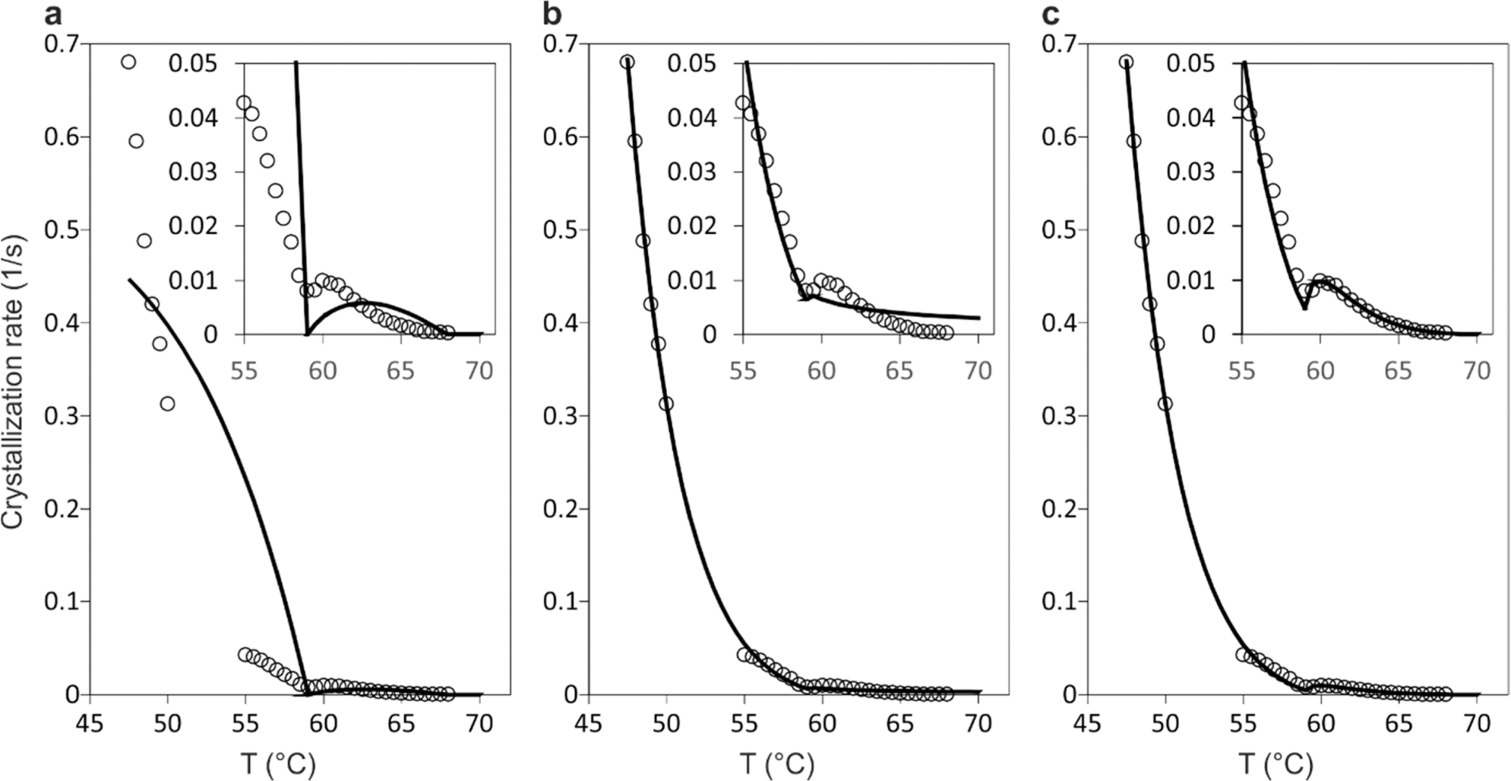
Comparison between best-fit theoretical growth rates to that of the experimental data for PEB90 between 45 and 70 °C (from DSC data above 54.5 °C and FSC data below 50 °C),^3^ subject to different choices of *T*-dependence of the fitting parameters. The melting points of 3- and 4-layer forms used are 59 and 68 °C for (a), 62 and 72 °C for (b), and 60 and 69 °C for (c). (a) Constant *A* and *A*′, *C* = 0. (b) Both *A* and *A*′ are exponentially proportional to crystallization driving force, with *C* remaining constant. (c) Both *A* and *A*′ are exponentially proportional to the crystallization driving force, and *C* is proportional to *A*.

In fitting the experimental data, we should also need to make assumptions about how *A*, *A*′, and *C* depend on temperature. These are determined by the free energy barrier of the corresponding growth step, and the simplest approach would be to assume that all three parameters are constant. However, the theoretical curve using constant values of *A* and *A*′ does not fit the experimental data well, particularly in the lower temperature part of the F3 growth branch. This is clearly shown in Figure 2a in the temperature region between 45 and 70 °C, where experimental data on PEB90 (DSC data above 54.5 °C, FSC data below 50.0 °C) and the best-fit theoretical curve assuming constant *A* and *A*′ and *C* = 0 are compared. With increasing undercooling, the experimental crystallization rate (circles) of F3 increases with acceleration, but if we keep *A*′ constant, the slope of the theoretical curve (solid line) decreases instead.

In an ultimately fine-grain theory, the elementary step would be the attachment of an individual CH_2_ group, and the height of the barrier would be independent of the driving force Δ*F* and hence of the undercooling. However, the elementary step in our approach is deposition of a whole monomer unit which contains over 10 flexible C–C bonds. Attachment of such a monomer involves releasing bulk crystallization energy as the monomer unit is “zippering up” and attaching to the growth surface. Thus, the actual barrier is lower than it would be if the whole monomer clicks in at once as a unit, and it becomes still lower as temperature decreases. The neglect of the “zippering” nature of the process is even more severe in the LH theory which treats the whole stem as a unit, resulting in the unphysical “δ*l* catastrophe”, the divergence of the lamellar thickness at large supercooling. The introduction of the “apportioning factor” ψ only partially alleviated the problem by pushing it to a lower temperature.^29,30^

Much better fit to the experimental data can be achieved (Figure 2b) if we assume that for attachment of an F*N* stem, with increasing undercooling Δ*T*_*N*_ = *T*_m,*N*_ – *T*, the free energy barrier decreases, and the decreasing Δ*F*_B,*N*_ is linearly proportional to the driving force for crystallization Δ*T*_*N*_ Δ*S* = (*T*_m,*N*_ – *T*)Δ*S* so that
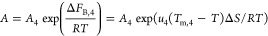
16a
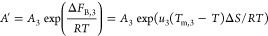
16bHere, *A*_4_ and *A*_3_ are the attachment rates of the F4 and F3 stems at the melting points of the respective forms, and *u*_4_ and *u*_3_ are constants (and fitting parameters) that link the decrease in free energy barrier to the corresponding driving force for crystallization. The assumption that the free energy barrier decreases with decreasing *T* can be justified by the fact that at larger supercooling, the stem does not have to be fully crystallized in order to be stable, i.e., a partial attachment already counts toward crystal growth. Therefore, it is understandable that the entropy barrier for a molten stem to extend sufficiently will decrease with decreasing temperature.

Without a conversion process (*C* = 0), referred to as “sliding diffusion” in ref (18), our model would predict zero growth rate at *T*_m,3_, i.e., crystal growth would stop completely due to self-poisoning, as shown clearly in Figure 2a and the inset. This was also shown by the rate-equation treatment of *n*-alkanes when the conversion process was omitted.^15^ In fact, complete cessation of crystal growth at the rate minimum was indeed observed in solution crystallization of alkanes at the transition from extended to once-folded growth,^7,8,11^ only to resume when a dilution wave reached the site reducing the solute concentration and with it the poison.^12^ The nearly zero conversion rate in solution crystallization is probably due to the molecular chains attaching at the growth front folded exactly in 2 (or 3, or other integer numbers), resulting in a high free energy barrier for their unfolding.

As a small but significant growth rate is still observed experimentally in PEB polymers at and around the growth rate minimum, a finite value of conversion rate *C* must be used. A constant *C*, however, reduces the growth rate of the F4 only at temperatures very close to the F4 to F3 transition (Figures 2b). It has also an additional problem that at higher temperatures, we arrive at an unrealistic situation that *C* > *A* (as *A* is decreasing with increasing crystallization temperature according to eq 16a), i.e., that conversion rate of covered stems exceeds the attachment rate *A*. Therefore, it is decided that having *C* as a fraction of *A* would be a more sensible choice. Indeed, this assumption leads to an almost perfect fitting of the experimental curve for PEB90 as shown in Figure 2c.
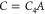
17

The fitting for the growth in the lower temperature range, between F3 and F2, can be carried out in the same way, except that *N* = 3 and we use the respective melting temperatures, *T*_m,3_ and *T*_m,2_. The equations can be written as
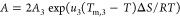
18a
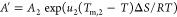
18b
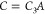
18cHere, the attachment rate *A* of the F3 stems (eq 18a) is related to the rate *A*′ of the F3 stems for the F4 to F3 transition, multiplied by a factor of 2. This is because the growth of an F3 stem at the higher temperature fitting (F4–F3) was modeled as a single step growth, while that in the fitting in the lower temperature range (F3–F2), the growth of an F3 stem takes two steps. However, because the attachment of an F3 stem happens through two consecutive substeps, not at the same time, the free energy barrier is the same. Therefore, the temperature dependence of the attachment rate stays the same too. With these restrictions added, we fit the overall curve for a polymer, with a weighted average for *A*_3_ and *u*_3_, and their initial values calculated from separate fittings in F4–F3 and F3–F2 temperature regions.

It should be mentioned that the best-fit *C*_3_ and *C*_4_ values (Table 2) are small, i.e., the conversion rate *C* is much smaller than attachment rate *A*. In fact, larger conversion rates would in general result in shallower growth rate minima. For example, in the MC simulation by Ma et al.,^18^ an artificially high chain-extension (or sliding diffusion) was imposed to make the simulation manageable, but the rate minima obtained were much shallower than in the experiments.

**Table 2:** Best-Fit Parameters to Experimental Growth Curves of PEB27, PEB90, and PEB188^a^

polymer	*u*_2_	*u*_3_	*u*_4_	*A*_2_ (/s)	*A*_3_ (/s)	*A*_4_ (/s) × 1000	*C*_3_ × 100	*C*_4_
PEB27	0.912	3.90	7.95	4.17	0.217	7.34	7.26	0.100
PEB90	0.611	5.71	9.01	11.0	0.143	2.34	3.97	0.174
PEB188	1.35	3.41	11.6	7.21	0.312	1.97	4.63	0.043

a *A*_2_, *A*_3_, and *A*_4_ are the attachment rates for F2, F3, and F4 forms at their respective melting points, and *u*_2_, *u*_3_, and *u*_4_ define the temperature dependence of the attachment rates on undercooling. *C*_3_ and *C*_4_ are the ratios between the conversion rates *C* and attachment rates *A*, for F2–F3 and F3–F4 conversion, respectively.

## IV Results and Discussion

**Figure 3:**
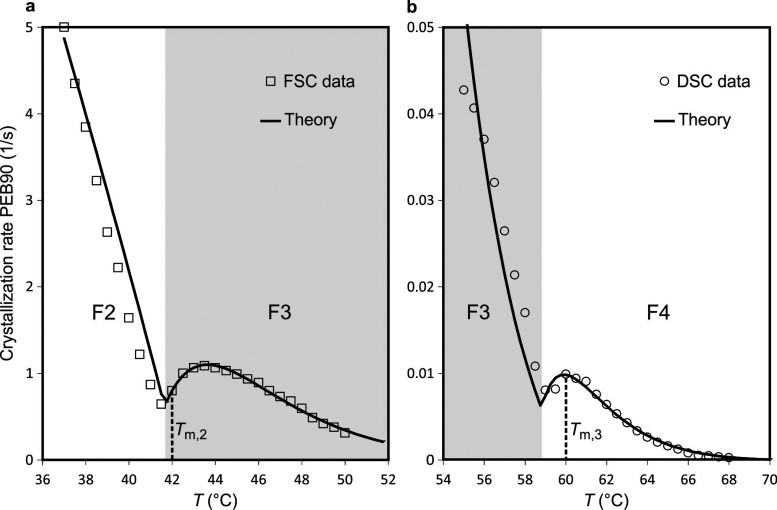
Individual fittings of experimental data. (a) Fit of the model to the growth curve between the F3 and F2 forms and (b) between the F4 and F3 forms of PEB90. The temperature regions for F3 growth are shaded in gray.

In an attempt to reproduce the experimental crystallization rate curves for each polymer, we have carried out the fitting for the high-temperature and low-temperature parts of the curves separately first. Experimentally these two parts are in fact measured using two different methods, conventional DSC for the upper part and FSC for the lower part, since crystallization at lower temperatures is too fast to be captured by conventional DSC. Each part of the curve can be further divided in two: above the rate minimum, where F4 (or F3) form grows, and below it where F3 (or F2) growth takes over. As the F3 (F2) form growth rate is determined only by *A*′ and *B*′ (eq 7), we have therefore used the low-*T* part of the curve first to find the best-fit parameters *A*_3_ (*A*_2_) and *u*_3_ (*u*_2_), before moving on to the high-*T* part of the growth curve to find the best remaining parameters. The results showing the very satisfactory fit of the theory to experimental data for the ranges around the two growth rate minima are shown in Figure 3.

**Figure 4:**
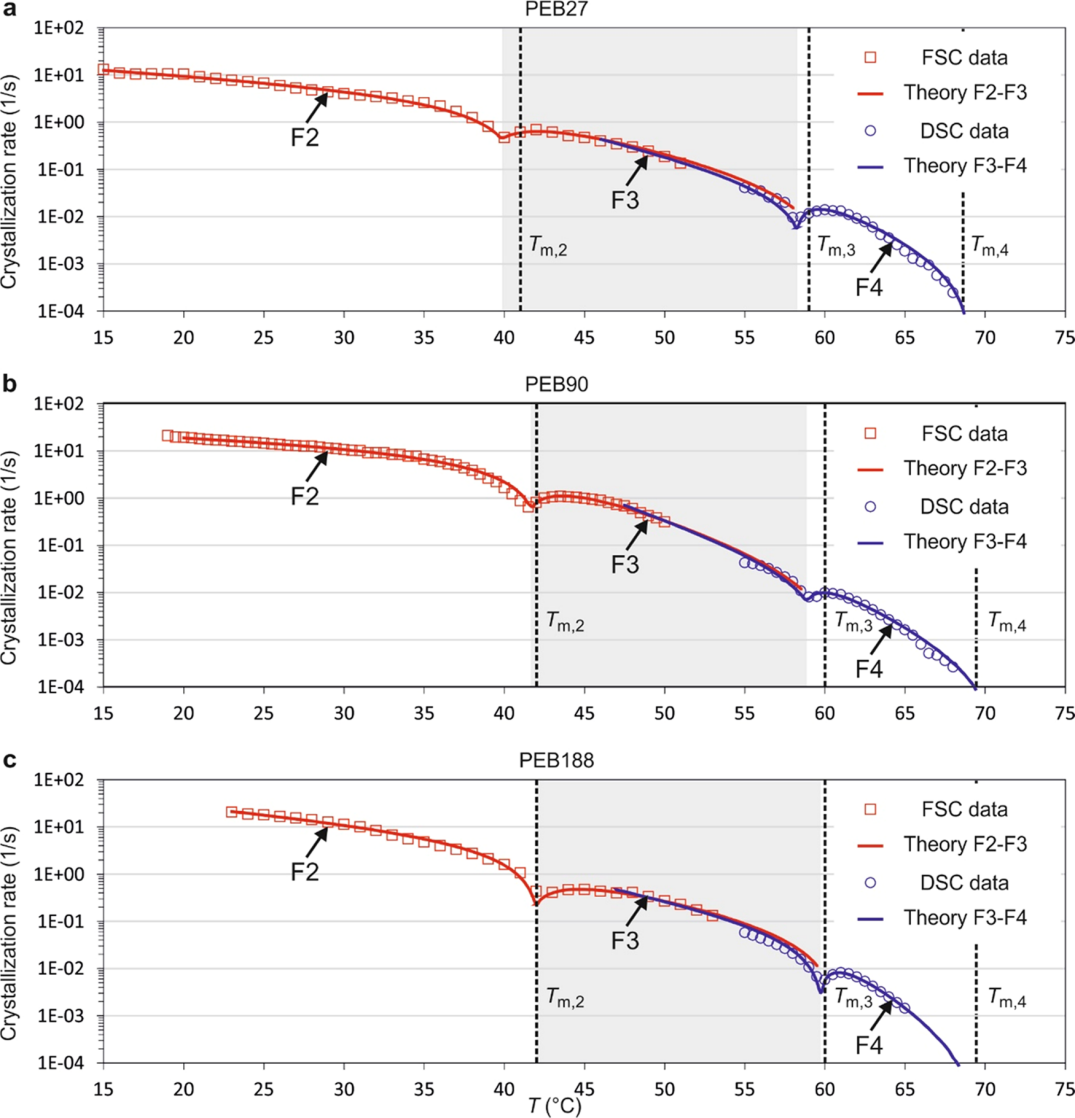
Experimental (squares and circles) and best-fit theoretical (solid curves) crystallization rate curves on a logarithmic scale. There are two theoretical curves for each polymer, one covering the growth of F4 and F3 forms and the other the growth of F3 and F2 forms. As for the blue curves, no conversion from F2 is considered for the growth of F3, leading to differences in the parameters used in the red and blue curves if fitted separately. Therefore, the same weighted averages of parameters *A*_3_ and *u*_3_ for the growth of the F3 form are used for the blue (F4/F3) and red (F3/F2) theoretical curves. The melting points of F4, F3, and F2 forms are shown by vertical dashed lines. Panels (a), (b), and (c) show the experimental crystallization rate and the two theoretical curves for the overall growth of PEB27, PEB90, and PEB188, respectively. The temperature regions for the growth of F3 form are shaded in gray.

The overall curve across the whole temperature range is then fitted by reconciling the parameters for the growth of the F3 for the low- and high-*T* ranges as mentioned before. The best-fit parameters are listed in Table 2, and the comparison of the experimental and overall simulated curves is shown in Figure 4.

Very good fittings to the experimental crystallization rate curves have been achieved based on our model, and that is evident in Figure 4 where the best-fit theoretical curves are compared to experimental ones. The melting points of the F(*N* – 1) forms are 1–2° higher than the temperature at which the local growth rate minimum is observed (*T*_min_). This is in fact what is expected when there exists a finite conversion rate *C*. Between *T*_m,*N*–1_ and *T*_min_, even though the F(*N* – 1) form is already stable relative to the melt, its “conversion front” to the F*N* form advances faster than the growth front of the F*N* form, so that only a finite number of F(*N* – 1) stems stand between the two fronts. Only below *T*_min_ will the F(*N* – 1) advance leave the conversion front behind and thus disable its progress.

In PEB, the two rate minima are quite far apart, meaning that the effect of F4 growth on the low-temperature range and that of F2 growth on the high-temperature range can be effectively ignored. Therefore, in each temperature region, fitting to the growth rate could be carried out separately. In the temperature range of the F3–F2 transition, poisoning of F3 growth by F2 attachment and conversion from F2 to F3 were included (Figure 4, red curves). Here, the fitting is good, despite the fact that potential F4 depositions were ignored. However, within the range of F3 growth, although we tried to reconcile the “high-temperature” (blue) and the “low-temperature” (red) fitted curves, they do not completely merge, even for the best-fitted polymer PEB90 (Figure 4b). Experimental error could have contributed to the discrepancy, as the two parts were measured using different methods (DSC and FSC, respectively). However, a contributory cause of the imperfect match at the F4 to F3 growth transition could be the fact that, while F3 growth by F2 attachment as well as conversion from F2 to F3 were included in the low-temperature (red) curves, they were not included in the high-temperature (blue) ones.

On closer examination of the fitting parameters listed in Table 2, with increasing molar mass, the attachment rate *A* for F4 decreases and shows stronger dependence on supercooling (larger *u*_4_) as expected. However, most other fitting parameters do not show such a clear trend. This is partly expected as the original data do not show a clear dependence on molar mass either. For example, the medium molar mass PEB90 shows the highest crystallization rate in F3 and upper F2 regions.^3^

It should be noted that the current model is highly simplified. For example, the growth of the polymer crystal is considered as a 1D process, the attachment/detachment of a monomer repeating unit is treated as a single thermodynamic step, and the details of the formation of the crystal–amorphous surface are ignored. The effect of nucleation (primary as well as secondary) and their possible different dependencies on supercooling are not considered either. However, satisfactory quantitative fitting to the experimental data has been achieved with reasonable fitting parameters, and the current model does have the advantage that it can be solved analytically. It would be desirable to develop a model in which the presence of all three different stem lengths could be taken into account at the same time, even though it is most likely that numerical methods would be required.

## V Conclusions

We have developed a simple theory that enabled us to explain quantitatively the multiple crystal growth rate minima observed in polymers with regularly spaced substituent groups. Our work confirms in a quantitative way the qualitative mechanism proposed by Marxsen et al. in their experimental work on PEB,^3^ also based on similar ideas in the previous studies on long alkanes.^11,14,15^ The reason behind the abnormal temperature dependence of the growth rate is confirmed to be self-poisoning resulting from temporary attachment to the growth surface of stems that are too short to be stable. Such a model will also contribute to a better general understanding of the complex process of polymer crystallization and identify and determine the key parameters controlling crystal growth rates. Since in more conventional polymers crystal layer thickness changes continuously with crystallization temperature *T*_c_, discrete rate minima are not seen. Nevertheless, self-poisoning is undoubtedly operative there too. At each *T*_c_ there is a minimum thickness *l*_min_ below which the crystal cannot grow, but this does not prevent stems slightly shorter than *l*_min_ attaching, lingering at the growth surface and obstructing productive growth with *l* > *l*_min_. Accordingly, some fine-grain simulations have shown evidence of such unstable stem attachments at the growth front.^18,21,22,31^ Consideration of the self-poisoning effect at a polymer growth front is needed for the development of a more realistic analytical theory of polymer crystallization. A considerable step in the right direction was made by Sadler with his roughness-pinning theory^21^ which, however, failed to reproduce most polymer crystal habits.

In addition to self-poisoning by stems of insufficient length, other types of poisoning effects, obstructing crystallization of polymers without impurities, are currently coming to light. The case of anomalous polypeptide concentration dependence on amyloid formation rate has already been mentioned.^20^ Recently, the well-recognized difficulty of crystallizing the desirable “stereocomplex” from racemic melt of L and D poly(lactic acid) has been attributed to “poisoning by purity”, i.e., to the rejection to the growth front of excess enantiomer resulting from concentration fluctuations.^32,33^ Recent preliminary work by our team also suggests that in some cases an unstable but kinetically favored polymorph could also hinder polymer crystal growth. Further work is in progress.
